# Retraction: Qian, W., et al. Efficacy of Chelerythrine Against Mono- and Dual-Species Biofilms of *Candida albicans* and *Staphylococcus aureus* and Its Properties of Inducing Hypha-to-Yeast Transition of *C. albicans*. *J. Fungi* 2020, *6*, 45

**DOI:** 10.3390/jof6020050

**Published:** 2020-04-18

**Authors:** Journal of Fungi Editorial Office

**Affiliations:** MDPI, St. Alban-Anlage 66, 4052 Basel, Switzerland; jof@mdpi.com

We have been made aware that the Figure 2 of the title paper [1] contains a genuine and serious mistake. The FESEM image of dual-culture (SA+CA) biofilms under the 1/2 MIC is a duplication of the image under 1/4 MIC. To ensure the addition of only high-quality scientific works to the field of scholarly publication, this paper [1] is retracted and shall be marked accordingly. The *Journal of Fungi* is a member of the Committee on Publication Ethics (COPE) and takes very seriously the responsibility to enforce strict ethical policies and standards.
